# Supplementary material for: Fast and reliable probabilistic reflectometry inversion with prior-amortized neural posterior estimation
Source: Sci Adv. 2025 Mar 14;11(11):eadr9668. doi: 10.1126/sciadv.adr9668 (PMC13109928; doi:10.1126/sciadv.adr9668)
Supplement: Supplementary file 1 — Figs. S1 to S9 [file sciadv.adr9668_sm.pdf]

Science Advances
AAAS

Supplementary Materials for

Fast and reliable probabilistic reflectometry inversion with prior-amortized neural posterior estimation

Vladimir Starostin *et al.*

Corresponding author: Vladimir Starostin, vladimir.starostin@uni-tuebingen.de;
Frank Schreiber, frank.schreiber@uni-tuebingen.de

Sci. Adv. **11**, eadr9668 (2025)
DOI: 10.1126/sciadv.adr9668

This PDF file includes:

Figs. S1 to S9

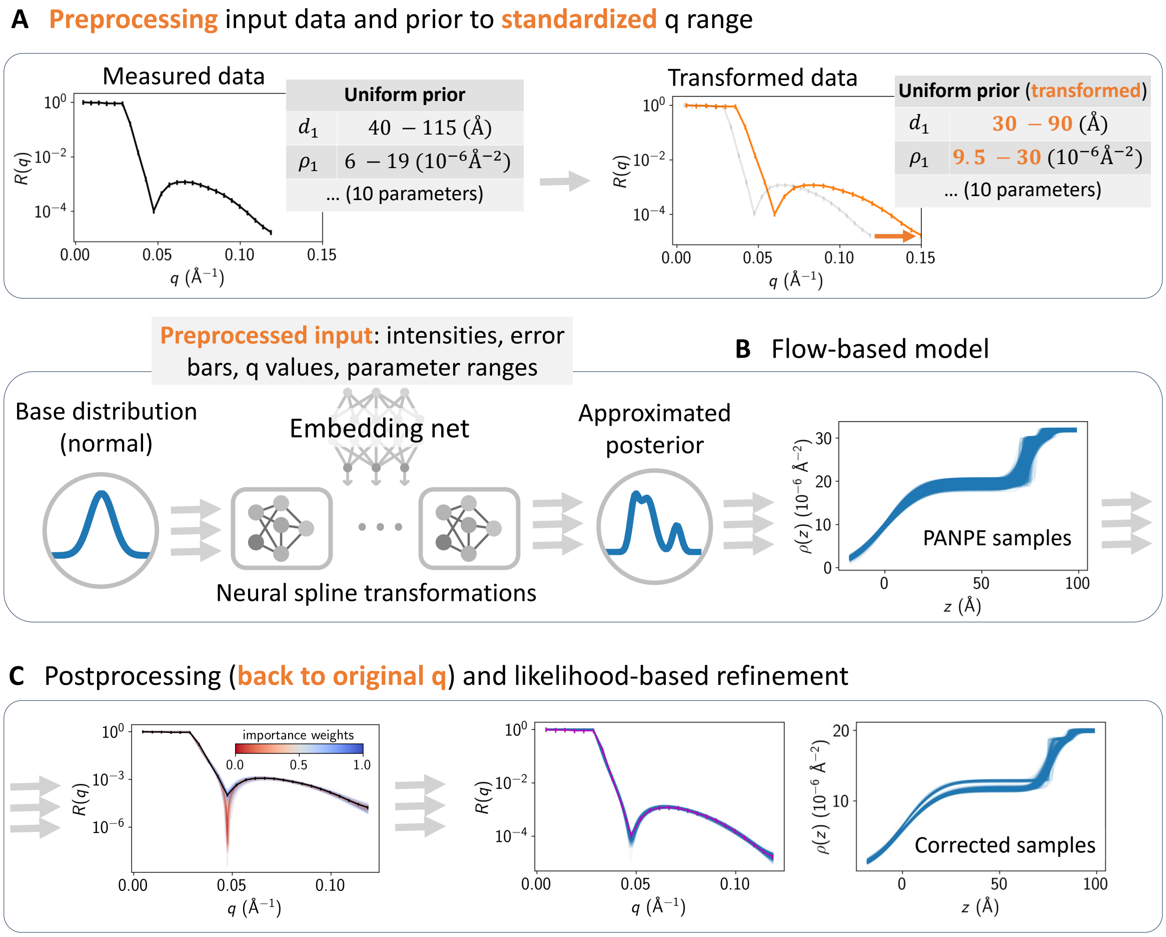

Figure 1: **Pipeline for PANPE-based Bayesian reflectometry analysis.** (**A**) The input data includes intensity values, q points and measurement uncertainties (error bars) along with the parameter ranges for each of the 10 parameters (for two-layer structures), that define the prior distribution. The data is first transformed to the standardized q range using equivariance in reflectometry data (see Methods). (**B**) The transformed data is supplied into the embedding network that supports varying number of q points. The resulting embedded data is provided to the normalizing flow model that transforms samples from the 10-dimensional standard normal distribution to the learned approximation of the posterior. The resulting PANPE samples are visualized as SLD profiles. (**C**) After transforming the sampled parameters back to the original q range, the reflectometry curves and the corresponding likelihoods are calculated. Likelihood evaluation enables likelihood-based refinement (importance sampling or MCMC) that provides the corrected samples.

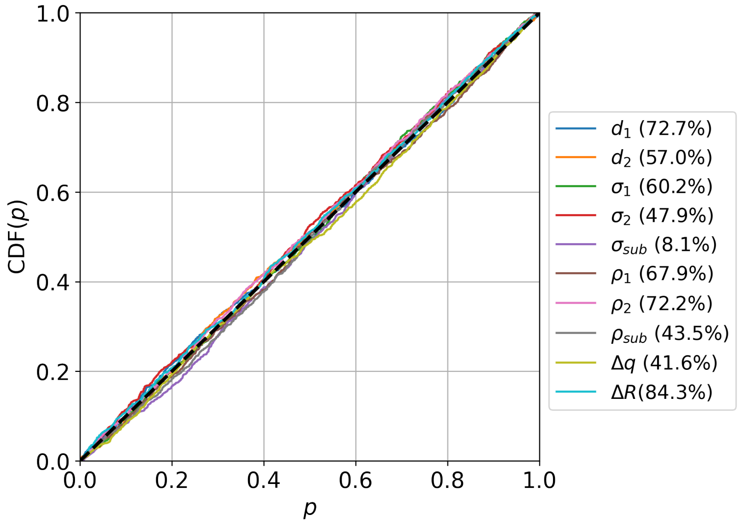

Figure 2: **P-P plots from the calibration tests on the simulated data.** One-sample Kolmogorov–Smirnov tests of our PANPE model for 10 marginal distributions performed on 1000 simulated test curves, with p values for individual parameters indicated in the legend.

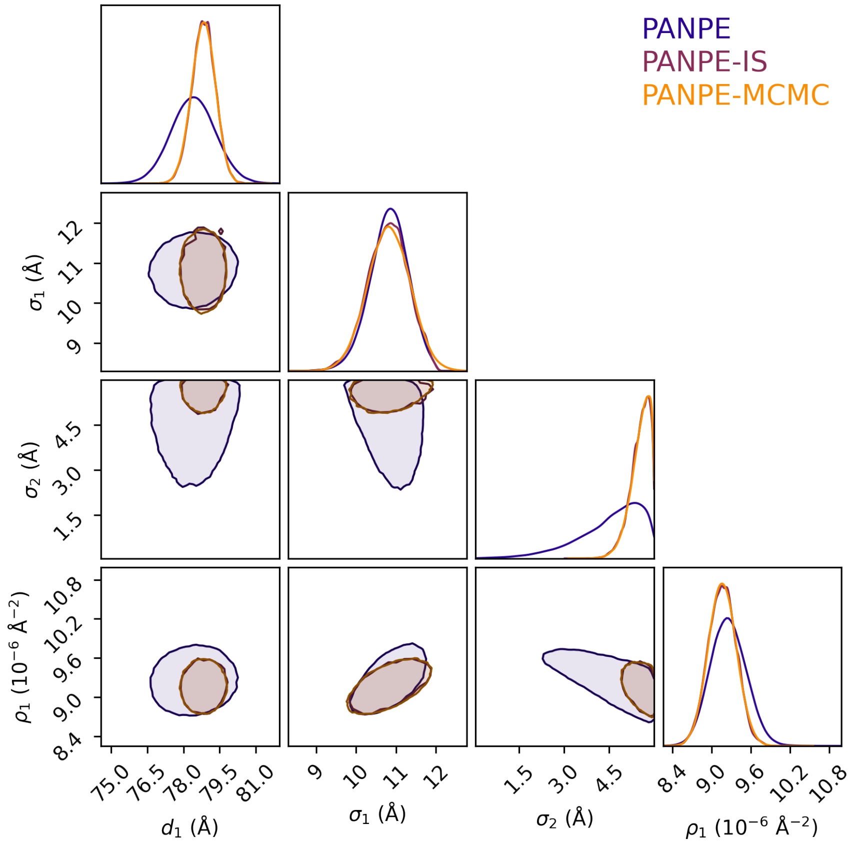

Figure 3: **PANPE results on a measured XRR curve**. The marginalized corner plot of 10-dimensional posterior distribution shows probability-mass covering proposal distribution provided by PANPE and two independently refined distributions via PANPE-IS and PANPE-MCMC, both of which yield equivalent solutions.

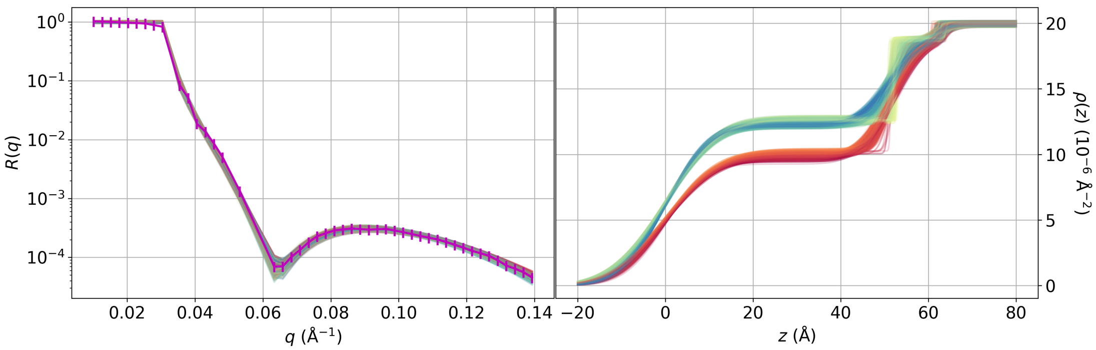

Figure 4: **An example of a multimodal solution on a measured XRR curve.** Reflectometry curves on the left include the measured curve, shown in magenta, alongside simulated curves corresponding to samples obtained through PANPE-IS, which colors match the colors of the respective SLD profiles on the right-hand side. The colors help recognise distinct SLD profiles.

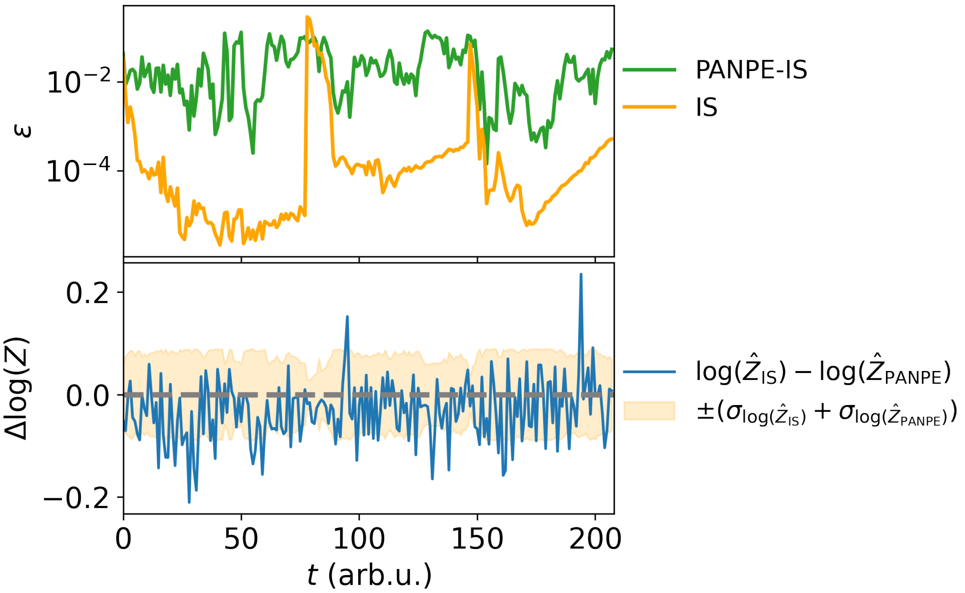

Figure 5: **Comparison between PANPE-IS and IS on the experimental data.** Top: sample efficiencies for conventional IS (orange) and PANPE-IS (green) on an experimental dataset of 208 X-Ray reflectometry curves. Here 3 datasets from real-time measurements are concatenated. Bottom: blue line is the difference between IS and PANPE-IS estimates of log evidence. The filled area denotes estimate errors.

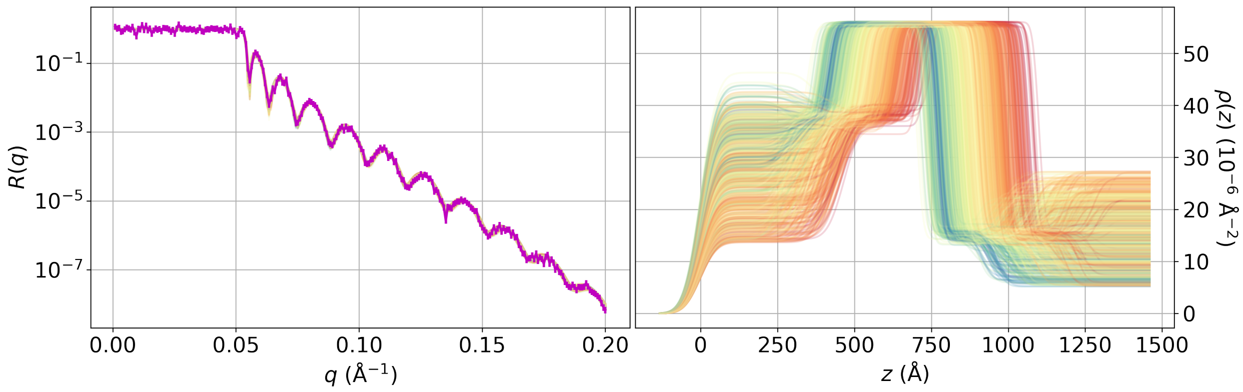

Figure 6: **Four-layer simulated structure analyzed by PANPE.** The PANPE-IS inference result for a simulated four-layer structure, which includes 16 parameters, demonstrates that increasing the number of layers can lead to highly ambiguous results. This necessitates the use of more prior information or additional measurements.

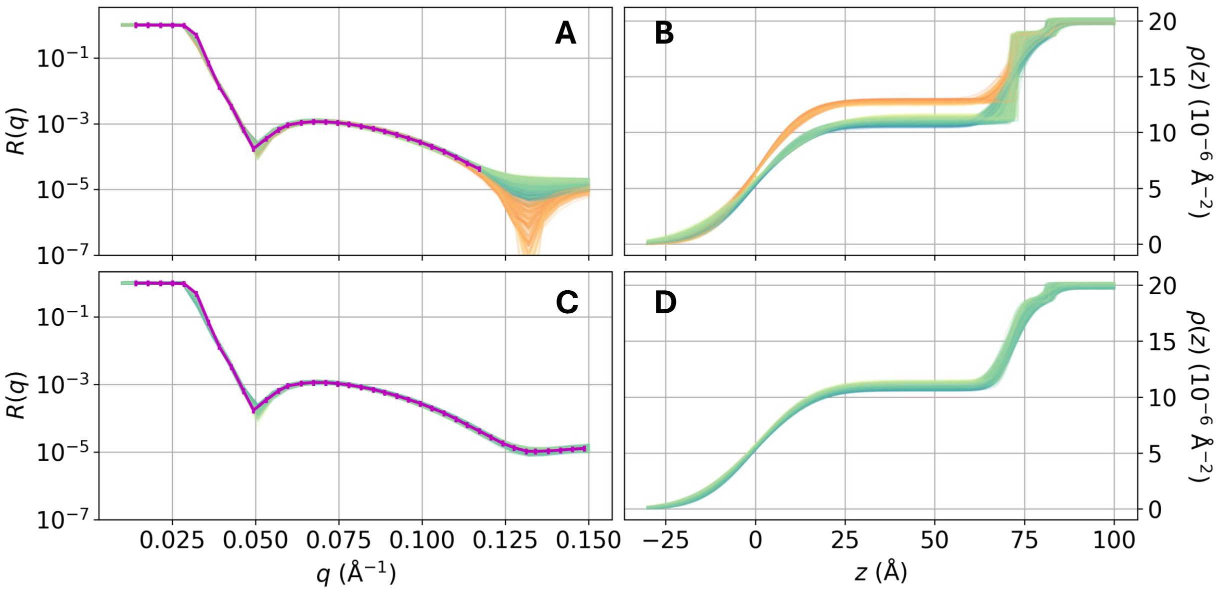

Figure 7: **Analysis of an experimental XRR curve performed at two measurement times.** (a) The experimental curve with 10 last q points retrospectively removed to simulate the data status at an earlier time in the measurement. (c) The complete measured curve. (b) and (d) display the corresponding SLD profiles sampled using PANPE-IS. In cases of ambiguity, such as between (a) and (b), simulated curves can guide the measurement to focus on the most informative q points (see the orange and green simulated curves in (a)). This approach can reduce the total number of required q points and speed up the measurements.

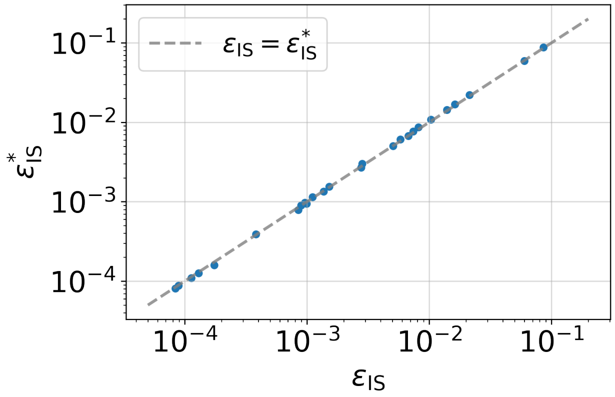

Figure 8: **The consistency of sample efficiency estimations.** The plot demonstrates the consistency between the standard sample efficiency estimations of the conventional importance sampling (ϵ_{IS} as per Equation 6) and via our model (ϵ^*_{IS} as per Equation 10) calculated for simulated samples with sufficiently high efficiency ϵ_{IS}.

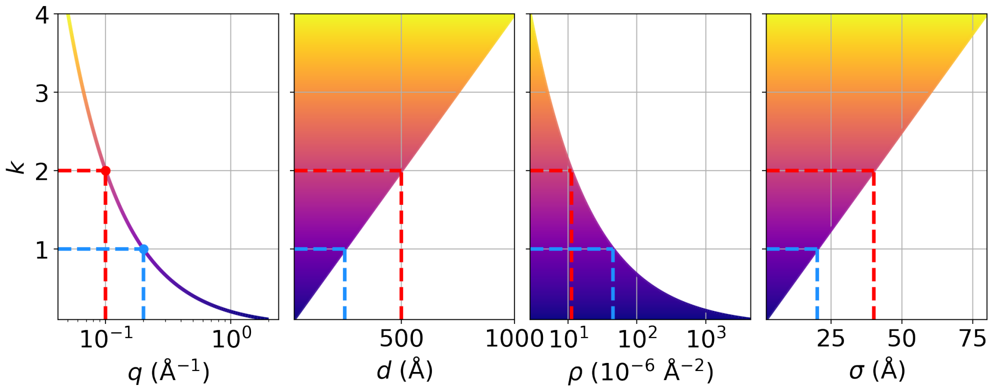

Figure 9: **Scaling invariance of the SLD parameters and momentum transfer q.** A model trained with a fixed q range $q_{max} = 0.2$ Å^{-1} (blue dashed lines) can be then applied to the measured data with a different q range, but the total parameter ranges are scaled accordingly. The red dashed lines demonstrate this scaling effect for $q_{max} = 0.1$ Å^{-1}. The colored areas denote the ranges within which the trained model can be applied.
